# High-throughput SNPs dataset reveal restricted population connectivity of marine gastropod within the narrow distribution range of peripheral oceanic islands

**DOI:** 10.1038/s41598-022-05026-z

**Published:** 2022-02-08

**Authors:** Daishi Yamazaki, Shun Ito, Osamu Miura, Tetsuro Sasaki, Satoshi Chiba

**Affiliations:** 1 Center for Northeast Asian Studies, Tohoku University, 41 Kawauchi, Aoba-ku, Sendai, Miyagi 980-8576 Japan; 2 Graduate School of Life Science, Tohoku University, 2-1-1 Katahira, Aoba-ku, Sendai, Miyagi 980-8577 Japan; 3 Faculty of Agriculture and Marine Science, Kochi University, 200 Monobe, Nankoku, Kochi, 783-8502 Japan; 4 Institute of Boninology, Chichijima-Aza-Nishimachi, Ogasawara, Tokyo 100-2101 Japan

**Keywords:** Population genetics, Biogeography

## Abstract

Molecular studies based on the high resolution genetic markers help us to grasp the factor shaping the genetic structure of marine organisms. Ecological factors linking to life history traits have often explained the process of genetic structuring in open and connectable oceanic environments. Besides, population genetic divergence can be affected by fragmented habitat, oceanic current, and past geographical events. In the present study, we demonstrated the genetic differentiation of marine gastropod *Monodonta* sp. within a narrow range of peripheral oceanic islands, the Ogasawara Islands. Genetic analyses were performed not only with a mitochondrial DNA marker but also with a high-throughput SNPs dataset obtained by ddRAD-seq. The results of the mtDNA analyses did not show genetic divergence among populations, while the SNPs dataset detected population genetic differentiation. Population demographic analyses and gene flow estimation suggested that the genetic structure was formed by sea level fluctuation associated with the past climatic change and regulated by temporal oceanographic conditions. These findings provide important insights into population genetic patterns in open and connectable environments.

## Introduction

Understanding the level of genetic differentiation and what factors caused it is a central issue in marine molecular studies^1,2^. In the open and connectable oceanic environment, several marine organisms tend to exhibit a high level of gene flow among populations^3,4^. For marine benthic organisms with low mobility in the adult phase, life history traits such as development types and the length of the planktonic larval phase are related to the level of dispersal ability and connectivity among populations. It often explains the process of genetic structuring in marine situations^5–8^. However, the length of the larval phase does not necessarily completely explain dispersal distance and genetic structure^9^. Accumulated molecular studies have demonstrated that other ecological characteristics such as habitat range and usage patterns are effective factors that cause population genetic differentiation^10–12^. Besides, the process of genetic population structuring is influenced not only by the above ecological factors but also by past geographical events, fragmented habitats and oceanic currents^13–15^. Climate fluctuation influences the population demographic history of various taxa, and genetic drift is a major factor in genetic differentiation^16,17^. After these events, it is predicted that temporal oceanographic conditions regulate dispersal via seawater and influence population genetic structures.

Delineating the genetic dynamics of marine species depends on the resolution of genetic markers. To detect the level of gene flow with a high degree of precision, molecular analyses using high-throughput data have been performed. For wild and non-model species, recent progress in genotyping methods through genome-wide variation of single-nucleotide polymorphisms (SNPs) have provided powerful tools, including restriction site-associated DNA sequencing (RAD-seq), double-digest RAD sequencing (ddRAD-seq), multiplexed ISSR genotyping by sequencing (MIG-seq), and genotyping by random amplicon sequencing (GRAS-Di). Our understanding of the process of genetic differentiation of various marine taxa is growing rapidly due to these remarkable genomic technologies^18–21^.

Oceanic islands, which are remote and isolated from continental landmasses, have been considered a suitable model for evolutionary studies^22^. Since the voyage of the HMS Beagle, many of Darwin’s followers have regarded oceanic islands as a kind of ‘evolutionary laboratory’ due to their outstanding species diversification, mainly in terrestrial taxa^23–26^. In addition, oceanic islands harbour distinct marine fauna composed of species that could reach and settle into these distant environments^27–29^. Some species isolated from their continental relatives have evolved to become endemic to the islands. Oceanic islands provide a simple model for studying the process of genetic differentiation following divergence from ancestral species.

The Ogasawara Islands, located in the northwestern region of the Pacific Ocean and approximately 1000 km from the Japanese mainland, are typical oceanic islands as they have never been connected to the Eurasian continent. The Ogasawara Islands comprise 30 islands that are categorized into four regions: the Mukojima Islands, Chichijima Islands, Hahajima Islands, and Volcano Islands. The terrestrial ecosystem of the Ogasawara Islands is unique and commonly referred to as an ‘Oriental Galapagos’, since it is characterized by a large number of endemic taxa^30–32^. Naturally, several marine and freshwater species have also successfully colonised the islands, some of which were investigated using molecular methods and identified as endemic, including fish, crabs, and molluscs^33–38^. These endemic species provide a suitable study system for investigating the factors shaping genetic structure in the Ogasawara Islands after divergence from their continental relatives. However, even though on the famous oceanic islands such as Hawaii and the Galapagos Islands, population genetic studies for endemic species were performed within the archipelago^28,39,40^, little is known about the Ogasawara Islands.

**Figure 1:**
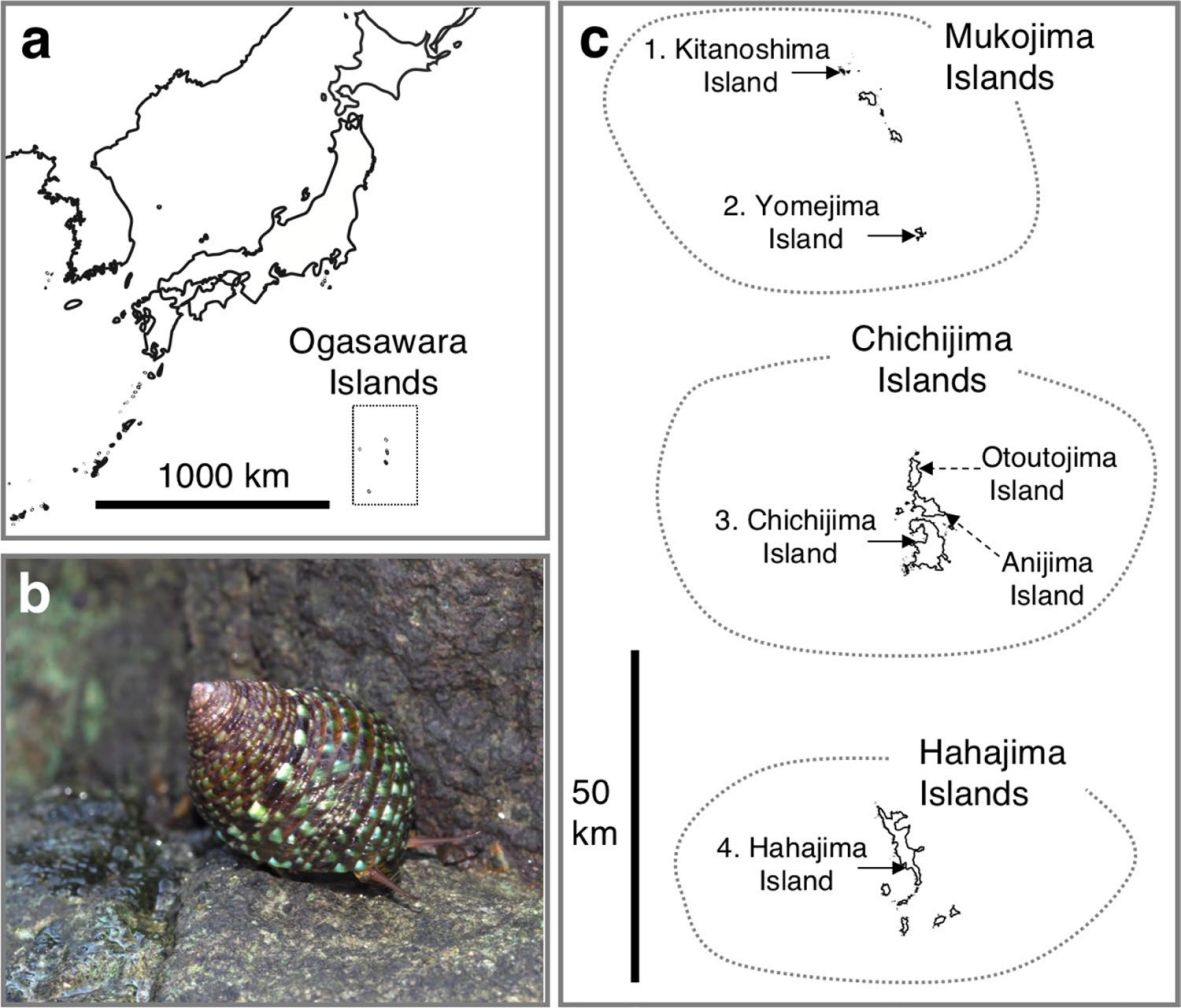
(**a**) Map showing the location of the Ogasawara Islands. (**b**) Photograph of living *Monodonta* sp. endemic in the Ogasawara Islands. (**c**) Map of the Ogasawara Islands. Island name with numbers (1–4) is the present survey localities.

In this study, we focused on an endemic marine snail species with a planktonic dispersal ability distributed in the intertidal zone of the Ogasawara Islands: *Monodonta* sp. (Fig. 1). This species has long been recognised as *M. australis* which is distributed in the Indo-Pacific region*.* However, a previous phylogenetic study demonstrated that *Monodonta* sp. distributed on the Ogasawara Islands is phylogenetically distinct from *M. australis* of Indo-Pacific^35^*.* Besides, the previous study demonstrated that *Monodonta* sp. is endemic to the Ogasawara Islands and derived from its Eurasian continental relative, *M. confusa*^35^. In the present study, we thus refer to this Ogasawara endemic *Monodonta* snail as *Monodonta* sp., not *M. astralis*. *Monodonta* sp. is common species and its distribution range is from the Mukojima Islands to the Hahajima Islands (< 120 km); it does not extend into the Volcano Islands, which constitute the southernmost region of the Ogasawara Islands^41^. The life cycle of *Monodonta* sp. on the Ogasawara Islands is not well understood, but knowledge of East Asian *Monodonta* species including *M. confusa*, which is a continental relative of *Monodonta* sp., is available^42–45^. According to these studies, *Monodonta* species undergo a planktonic larval duration of 3 days (*M. confusa* and *M. perplexa*). After larval settlement and metamorphosis, *M. confusa* matures to a shell height of approximately 10 mm, which takes 1 to 2 years. Around the Japanese archipelago*,* a previous molecular study on *M. confusa* detected a significant genetic population structure, but the level of differentiation was smaller than that of closely related species^46^. For *Monodonta* sp. distributed in the Ogasawara Islands, previous phylogenetic analyses based on standard molecular markers showed no differentiation within the Ogasawara Islands^35^. However, given the relatively short planktonic larval duration of *Monodonta*, it is possible that *Monodonta* sp. is genetically differentiated across the archipelagos. To clarify the genetic structure of marine species within the Ogasawara Islands, it is necessary to carry out a high resolutive molecular approach.

Here, we aimed to determine the level of genetic differentiation and patterns of genetic structuring within a narrow range of peripheral oceanic islands using marine organisms *Monodonta* sp*.* as our model. In addition, we tested the hypothesis that genetic differentiation occurred due to historical climatic events and regulated temporal oceanographic conditions. Genetic analyses were performed using a mitochondrial DNA (mtDNA) marker, and a high-throughput SNPs dataset obtained by ddRAD-seq. Based on the dataset obtained by these two types of genetic markers, we discuss the genetic structure, temporal population connectivity, and timing of genetic divergence in open and connectable marine environments of peripheral oceanic islands.

## Results

### Genetic structure estimated by mtDNA datasets

**Figure 2:**
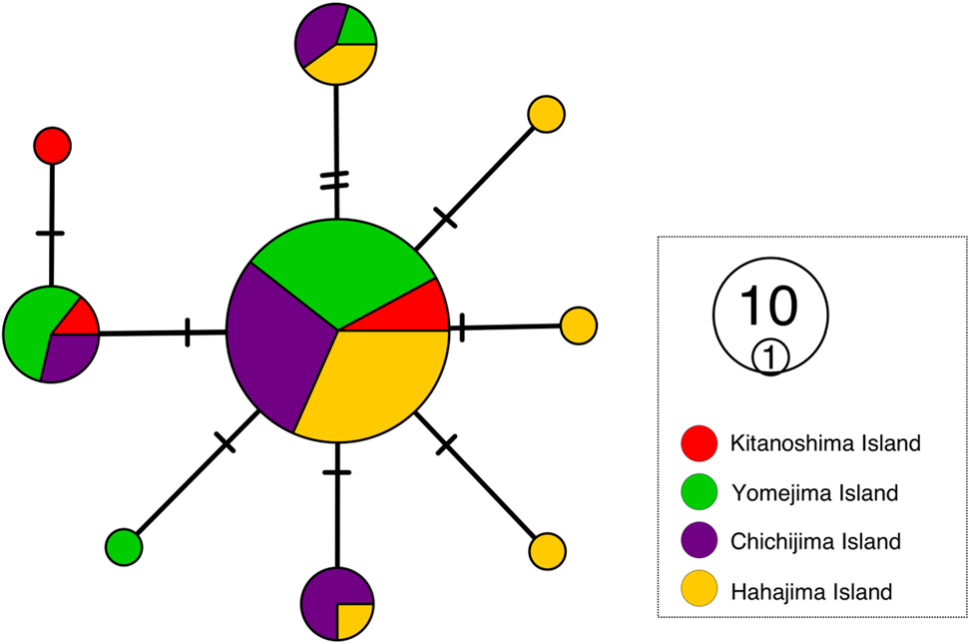
Haplotype network of *Monodonta* sp*.* reconstructed by using mtDNA.

The obtained length of the COI sequence alignment was 513 base pairs*. Monodonta* sp. had nine haplotypes from 59 individuals of four sampling islands. The haplotype diversity and nucleotide diversity of each population are shown in Table 1. The haplotype diversity ranged from 0.53 (locality 2: Yomejima Island) to 0.70 (locality 1: Kitanoshima Island), and the nucleotide diversity ranged from 0.00136 (locality 2) to 0.00195 (locality 1). The AMOVA analysis did not show the existence of population genetic differentiation in *Monodonta* sp. (*Φ*_ST_ = 0.02, *P* = 0.23; Table 2). Similarly, no genetic differentiation was detected in any combinations of pairwise *F*_ST_ values among the four populations (Table 3a). The haplotype network had a typical star-like shape, with one major haplotype (Fig. 2) and did not discriminate among localities.

**Table 1:** Sampling localities and population genetic diversity indices (COI gene) of *Monodonta* sp.

No	Sampling locality	nN	mtN	nH	HD	ND	IC
1	Kitanoshima Island	5	5	3	0.700	0.00195	0.001
2	Yomejima Island	8	18	4	0.529	0.00136	0.139
3	Chichijima Island	8	18	4	0.608	0.00180	0.499
4	Hahajima Island	8	18	6	0.562	0.00168	0.145

nN: number of individuals used data of nDNA obtained by ddRAD-seq analyses; mtN: number of individuals used mitochondrial population genetic analyses (COI); nH: number of haplotypes (COI); HD: Haplotype diversity (COI); ND: Nucleotide diversity (COI); IC: inbreeding coefficient estimated by using nDNA.

**Table 2:** Analysis of molecular variance (AMOVA) of *Monodonta* sp. using two kinds of genetic datasets (mtDNA snd nDNA).

	Source of variation	Fixation index	Percentage of variation
mtDNA	Among population	0.019	1.89
	Within population	(*P* = 0.229)	98.11
nDNA	Among population	0.219	21.91
	Within population	(*P* < 0.0001)	78.09

**Table 3:** Pairwise *F*_ST_ among populations of *Monodonta* sp. using (a) mtDNA and (b) nDNA.

Locality number	1	2	3
**(a)**			
2	− 0.021	–	
3	0.057	0.005	–
4	0.122	0.034	− 0.018
**(b)**			
2	0.327**	–	
3	0.357**	0.048*	–
4	0.378**	0.089**	0.025

*: *P*-value < 0.05; **: significant after Bonferroni correction.

### Genetic analyses based on the SNPs datasets

**Figure 3:**
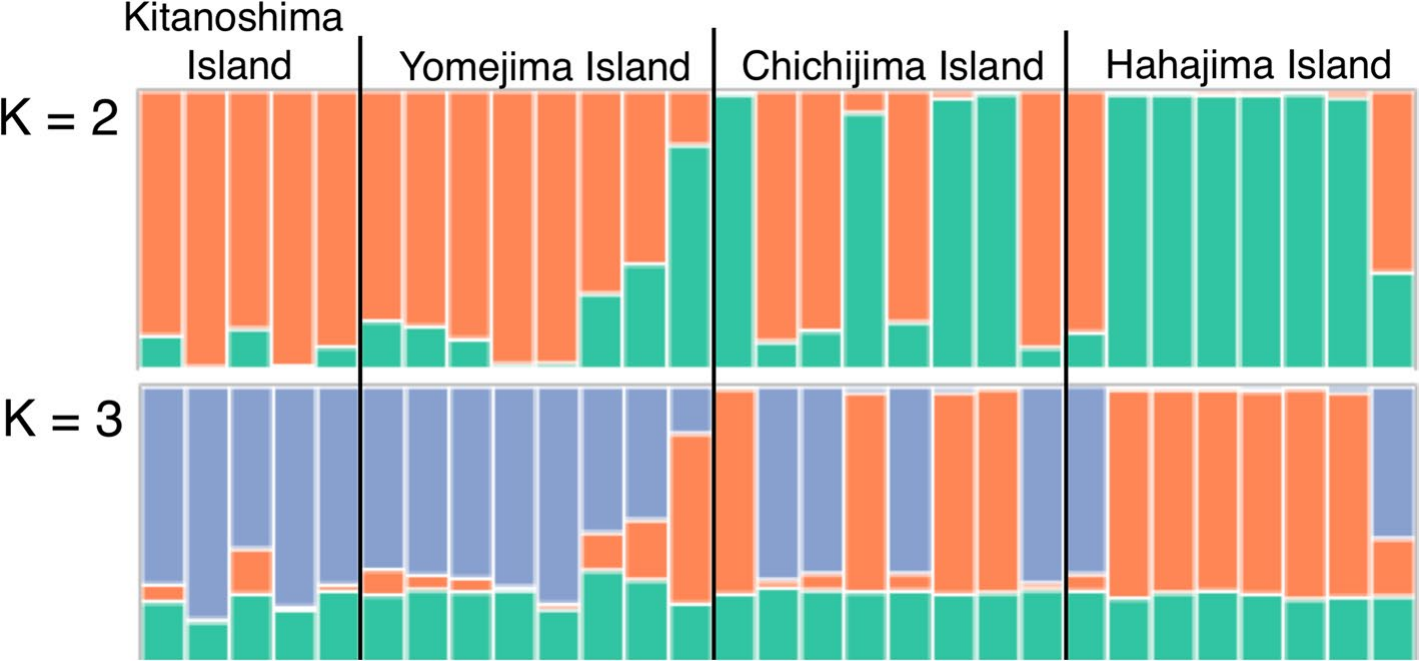
Results of the STRUCTURE analyses for K = 2 and 3 inferred from nDNA.

After demultiplexing the raw datasets, we obtained 675,370–10,388,536 reads for each individual. A total of 1731 filtered loci (9.72% of missing sites) and a total alignment length of 158,328 bp (9.20% of missing sites) were obtained (details in Table S1). The results of the STRUCTURE analysis are shown in Fig. 3, and the maximum value of Δ*K* was at *K* = 2. These two genetic clusters were mainly divided by the Mukojima Islands [Kitanoshima Island (locality 1) and Yomejima Island (locality 2)] and the southernmost Hahajima Island (locality 4). On Chichijima Island (locality 3), which is located centrally in the Ogasawara Islands, the two genetic clusters were mainly mixed. A scatterplot of the PCA also showed that individuals fell into two genetic clusters (Fig. S1). This trend was similar to the results of the aforementioned STRUCTURE analysis. In PCA plots [Fig. S1a (PC 1 and PC 2), b (PC 1 and PC 3)], the northern Mukojima Islands (locality 1 and 2) and several individuals of Chichijima Island and Hahajima Island came together in a single cluster. Other individuals of Chichijima Island and Hahajima Island formed a distinct cluster. The above two clusters did not appear in PC2 and PC3 (Fig. S1c).

**Figure 4:**
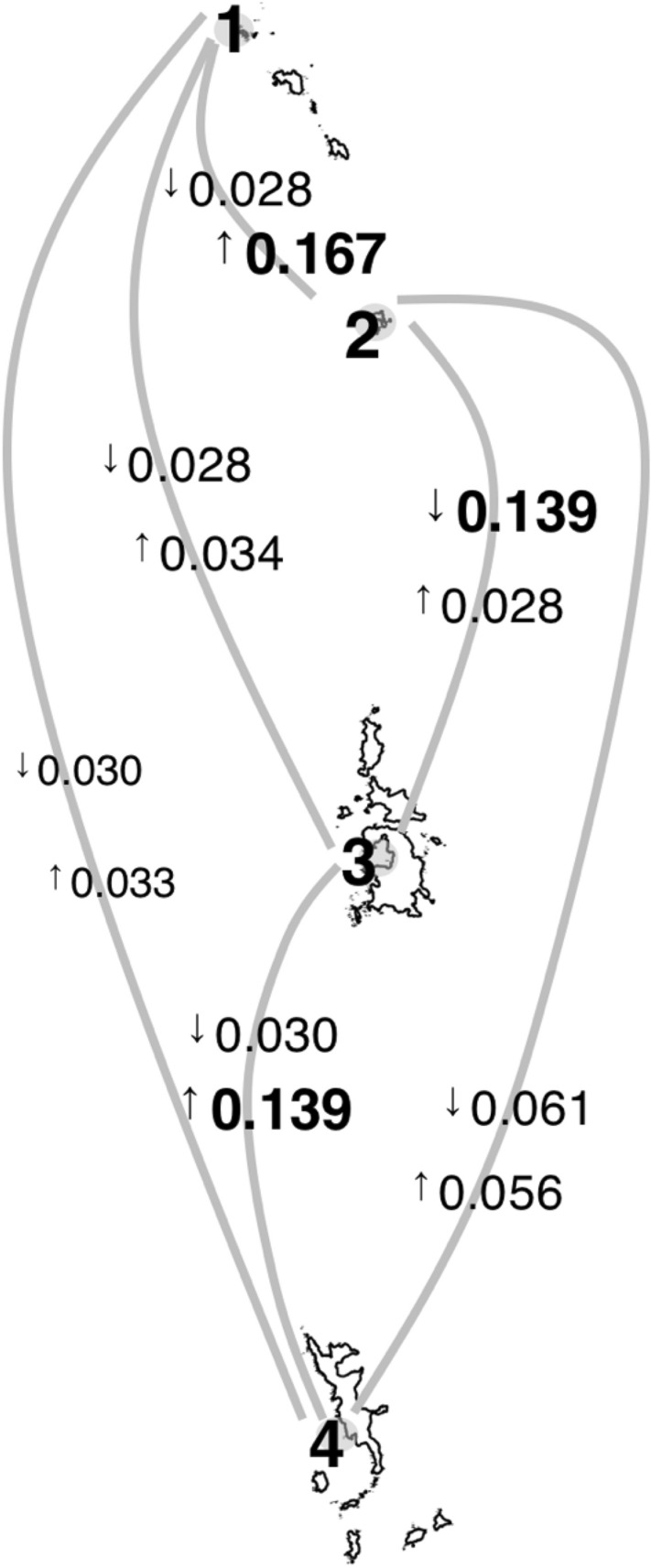
Results of Bayesian estimation of temporal gene flow among four populations of *Monodonta* sp*.* within the Ogasawara Islands. The numbers on the map mean locality number of population (1: Kitanoshima Island; 2: Yomejima Island; 3: Chichijima Island; 4: Hahajima Island). The numbers above the line connecting two populations indicate the proportion of gene flow and the arrow mean direction of it.

The BA3-SNPs analysis indicated the gradient of gene flow among populations (Fig. 4), which did not necessarily correspond to the geographic distance between islands. Migrations from Yomejima Island and Hahajima Island to Chichijima Island tended to occur relatively easily (> 0.10), while those from Chichijima Island to other islands were less likely to occur (< 0.04). Migrations between Yomejima Island and Hahajima Island were infrequent. The inbreeding coefficient exceeded 0.10 at each locality except at Kitanoshima Island, and was about 0.5 at Chichijima Island (Table 1).

The AMOVA estimated by using these SNPs dataset showed the presence of population genetic differentiation (*Φ*_ST_ = 0.219, *P* < 0.0001; Table 2). Pairwise *F*_ST_ values between the populations were higher than the results from mtDNA (Table 3). Except for pairwise *F*_ST_ values between Chichijima Island and Hahajima Island, genetic differentiation was detected between the population.

**Figure 5:**
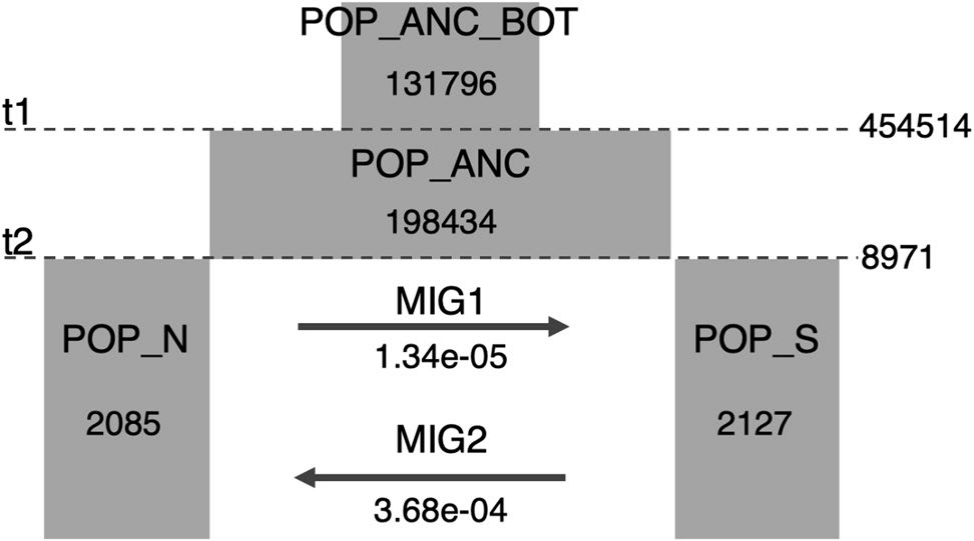
Estimation model of genetic differentiation of *Monodonta* sp. in the Ogasawara Islands. POP_ANC_BOT: ancestral population bottlenecked arriving the oceanic Ogasawara Islands; POP_ANC: ancestral population; POP_N: northern population (Kitanoshjima Island and Yomejima Island); POP_S: southern population (Chichijima Island and Hahajima Island); MIG1: migration rate from POP_N to POP_S; MIG2: migration rate from POP_S to POP_N.

The estimating the timing of genetic differentiation by fastsimcoal2 approach showed that the northern (Kitanoshima Island and Yomejima Island) and the southern (Chichijima Island and Hahajima Island) islands are genetically differentiated at 8971 generations ago (t2) (Fig. 5). The recovery timing of ancestral population size from the bottleneck that occurred when arriving at the Ogasawara island is at 454,514 generations ago (t1).

## Discussion

The present study tested the level of population genetic differentiation of an intertidal gastropod species distributed within a narrow range of peripheral oceanic islands using two types of genetic markers. A genome-wide single nucleotide polymorphisms (SNPs) dataset obtained by double-digest RAD sequencing (ddRAD-seq) was able to detect genetic differentiation among populations of *Monodonta* sp. endemic in the Ogasawara Islands. In the case of our study, the mitochondrial DNA (mtDNA) did not provide sufficient information on genetic polymorphisms compared to the above high resolution genetic marker. For molecular ecological studies, mtDNA has been commonly used to demonstrate the population genetic structure and evolutionary history of various taxa. However, mitochondrial-specific traits such as frequent introgression may bias the investigation of population genetic structuring and dynamics^47^. Incomplete lineage sorting also makes the interpretation of the genetic results difficult. The discordance often occurs between mtDNA and nuclear DNA (nDNA) due to their different inheritance patterns^48,49^ and mtDNA seems to lose the ancestral polymorphisms faster than nDNA^50,51^. In contrast, genetic traces left in mtDNA are important for understanding the complex evolutionary history between organelle and nuclear genomes^52^. By using both organellar DNA and genome-wide datasets, we can understand the population genetic dynamics in detail. From the above context, the present mtDNA results provided useful information on the differences in genetic diversity between *Monodonta* sp. on the Ogasawara Islands and its closely related continental species, *M. confusa*. The mtDNA of *M. confusa* displays a higher level of genetic diversity around the Japanese archipelago and the continental side than *Monodonta* sp.^46,53^. This reduced genetic diversity of *Monodonta* sp. observed in mtDNA is attributed to the effects of bottlenecks, and similar patterns have been detected in other organisms of the Ogasawara Islands^54^. These results indicate that endemic marine species underwent a strong bottleneck when colonising the Ogasawara Islands, which is considered a fundamental pattern in peripheral oceanic islands^28,40^.

Populations of *Monodonta* sp. accumulated genetic differentiation within a relatively narrow range from Kitanoshima Island to Hahajima Island (< 120 km). The genetic components of *Monodonta* sp. are divided between the Mukojima Islands (Kitanoshima Island and Yomejima Island) and the southern islands (Chichijima Island and Hahajima Island). The genus *Monodonta* is known to undergo a planktonic larval phase during its life cycle. In the case of *M. confusa*, which is the closest continental relative to *Monodonta* sp. endemic to the Ogasawara Islands, the planktonic duration is 3 days^45^. Besides, trochid snail species related to *Monodonta* have relatively short PLD (*Gibbula umbilicalis* and *Osilinus lineatus* ≤ 7 days)^55^*.* Dispersal ability during the planktonic larval phase is thought to be one of the most important factors for genetic differentiation among populations in the marine environment. However, a theoretical study indicated that population genetic connectivity is not necessarily determined by planktonic larval dispersal duration^56^. Isolated island populations have experienced significant founder effects and bottlenecks when colonising and could not receive migrants from the source continental populations. The genetic drift is thus important for the genetic differentiation of island endemic species^57,58^.

The geographical distance at which genetic differentiation occurs depends on the oceanographic conditions and specific characteristics of species^59^. Although the Kuroshio Current and its countercurrent are effective factors in the dispersal of marine organisms distributed in the North Pacific ocean^60^, the detailed system of oceanic currents around the Ogasawara Islands has not been sufficiently studied^61,62^. In seed dispersal plants *Pandanus boninensis* (Pandanaceae) distributed in the Ogasawara Islands, molecular studies with gene flow estimations demonstrated that migration mainly occurs from the south islands (Hahajima Island) to the north islands (Mukojima Island) via Chichijima Island and also from the Mukojima to the Chichijima Islands^62^. The result of gene flow estimation of *Monodonta* sp. also showed that the opportunities for migration to Chichijima Island from Yomejima and Hahajima Islands tended to occur relatively frequently, while reverse migration occurred less frequently. Although migration via oceanic currents vary among taxa around the Ogasawara Islands, there may be currents heading toward Chichijima Island from the south and north islands. In addition, frequent migration to Chichijima Island may also be due to the habitat preference of *Monodonta* sp. While *Monodonta* sp*.* is common in the rocky intertidal zone of the Ogasawara Islands, their inhabitation is less common in environments that are exposed to strong waves^63^. Chichijima Island may hold many suitable habitats for *Monodonta* sp*.* since it is the largest of the Ogasawara Islands and has a long coastline and large bay. Furthermore, the Chichijima Islands are composed of three large islands (Fig. 1; Otoutojima Island, Anijima Island, and Chichijima Island), and each island can weaken the effects of waves on the intertidal zone. In fact, the inbreeding coefficient of Chichijima Island is at a high level. The presence of such suitable habitats may assist in the settlement of larvae that migrate from other islands to the Chichijima Islands. *Monodonta* sp. is not distributed to islands surrounded by coastlines with strong wave exposure, such as Nishinoshima Island (approximately 130 km from Chichijima Island) and Kita-iwoto Island (approximately 150 km from Hahajima Island)^64,65^. This suggests that planktonic larvae of *Monodonta* sp. are unable to settle in an environment attacked by strong waves or it may be difficult to disperse over distances of 100 km.

The life cycle of *Monodonta* sp. on the Ogasawara Islands is not well understood, but the knowledge of *M. confusa* can be applied to estimate the results of population demographic estimation^42–44^. According to these studies, *M. confusa* matures in 1 to 2 years. If we apply the above generation time to interpreting the population demographic estimation of *Monodonta* sp. on the Ogasawara Islands (Fig. 5), the bottlenecked ancestral population size have recovered 454,514–909,028 years ago (t1). Therefore, the time of divergence of *Monodonta* sp. from its continental sister species is considered to be before t1, and there is no significant discrepancy between t1 and the divergence time of *Lunella ogasawarana*, which is an endemic turbinid gastropod of the Ogasawara Islands (1.0–2.7 Ma)^66^. At 8971–17,942 years ago (t2), northern (Mukojima Islands, including Kitanoshima Island and Yomejima Island) and southern populations (Chichijima and Hahajima Islands) started to become genetically divergent. In the late Pleistocene period, the shoreline was longer and the range of the intertidal zone (suitable habitat for *Monodonta* sp.) was wider, because the last glacial maximum (LGM, approximately 20,000 years ago) caused a drop of approximately 100 m in sea level^67^. During the LGM period on the Ogasawara Islands, the islands constituting the Mukojima Islands (e.g. Mukojima Island and Nakoudojima Island, except for Yomeshima Island) were connected. Similarly, the islands constituting the Chichijima Islands (e.g. Chichijima Island, Anijima Island, and Otoutojima Island), and the islands constituting the Hahajima Islands (e.g. Hahajima Island, Imotojima Island, and Anejima Island) were connected, respectively^68^. Following LGM, because the expansion of the intertidal zone was completed and the sea level began to rise, genetic differentiation of *Monodonta* sp. may have been initiated. Although the present pattern of genetic structure, which includes two different genetic components in the two most external islands and a mixture of the two in the central islands, seem to fit the stepping stone model, it is not confirmed by gene flow estimation. Therefore, this pattern was established by genetic differentiation between the north and south islands due to the above discussed past climate event and is thought to be regulated by the oceanic current moving to Chichijima Island.

In addition, population genetic differentiation of endemic species may be promoted by the disadvantages of dispersals in remote environments like oceanic islands isolated from the continental landmass^69–71^. Larval dispersal is considered to have several advantages, including colonising new areas and decreasing the likelihood of inbreeding and extinction due to genetic exchanges between populations^69,71–73^. Dispersal also fundamentally has various unavoidable risks. Even if *Monodonta* sp. maintains similar dispersal ability of its continental relatives, the dispersed larvae may not be able to settle in suitable habitats on isolated oceanic islands. If few suitable habitats are available, dispersed larvae may fail to settle successfully, thereby increasing mortality^74^. For the intertidal snail species genus *Littorina*, direct developing species are present on isolated and remote islands, while there are no closely related species with a pelagic larval stage (The paradox of Rockall)^75^. The possibility that isolated situations may promote genetic differentiation due to the disadvantages of larval dispersal is an interesting issue to be solved by future ecological studies on larval behaviours.

The marine species inhabiting the Ogasawara Islands are mainly characterised by taxa that once successfully colonised these islands after long-distance travel. Endemic phenomena in oceanic islands are affected by the distance of the island and time elapsed since colonisation^27^. Around the Ogasawara Islands, several marine species were confirmed to be endemic^33–38^. However, the level of genetic connectivity and differentiation within the Ogasawara Islands is poorly understood in other marine species. In the case of coral species with far-reaching dispersal ability, a low level of genetic differentiation among the three populations on the Ogasawara Islands was detected by microsatellite markers^76^. In contrast, *Monodonta* sp., with a relatively short larval phase, exhibited a genetic structure within this narrow range of archipelagos. Similarly, other endemic marine species with short dispersal ability may also show genetic differentiation within a similar range. To understand the connectivity among populations of the Ogasawara Islands, comparative genetic studies using multiple taxa are needed. The present study indicated that it is necessary for marine conservation on the Ogasawara Islands to focus on both the north and south island populations which differ genetically. Besides, the genetic structure of marine species may be more differentiated than previously thought within the Ogasawara islands. High resolution genetic analyses of various marine taxa will provide invaluable insights into marine conservation strategies in highly endemic water regions such as oceanic islands^77,78^. By clarifying the genetic structure and population demographic history of various species, we can set up a marine reserve system at an appropriate geographic scale^79,80^.

In conclusion, we detected the population genetic divergence of intertidal gastropod with a planktonic larval phase, even within a narrow range of the Ogasawara Islands. This was caused by historical climatic events and maintained by temporal oceanographic conditions. To accurately grasp the degree of genetic differentiation and population history, high-throughput genetic data is useful. In the genomic era in which we live, unexpected genetic differentiation is bound to be detected. These findings provide important insights for understanding the population genetic patterns of open and connectable environmental situations observed in remote oceanic islands.

## Methods

### Sample preparation

In total, 59 samples of *Monodonta* sp. were collected from the four localities of the Ogasawara Islands (Fig. 1; Table 1). Our sampling range covered the distribution area of *Monodonta* sp. on the Ogasawara Islands. To prepare the samples, a part of the foot muscle was dissected from each individual and stored in 99.5% ethanol for subsequent molecular analyses.

### Mitochondrial DNA sequencing and population genetic analyses

Total DNA was obtained from tissue samples using the NucleoSpin Tissue (TaKaRa, Shiga Pref., Japan) according to the manufacturer’s instructions. Fragments of the COI gene were amplified using the primers CoxAF (5′-CWAATCAYAAAGATATTGGAAC-3′) and CoxAR (5′-ATATAWACTTCWGGGTGACC-3′)^81^. Polymerase chain reactions (PCR) were performed under the following conditions: 94 °C for 3 min followed by 5 cycles at 94 °C for 30 s, 45 °C for 30 s, 72 °C for 1 min, followed by 35 cycles at 94 °C for 30 s, 52 °C for 30 s, and 72 °C for 1 min, with a final extension at 72 °C for 5 min. The products were then purified using ExoSAP-IT (Amersham Biosciences, Buckinghamshire, UK). Cycle sequencing was performed using the PCR primers with the BigDye Terminator Cycle Sequencing Ready Reaction Kit (Applied Biosystems, CA, USA) and the products were directly sequenced from both directions using an ABI 3130xl automated sequencer (Applied Biosystems). The validity of the sequences was verified with the software package 4Peaks^82^, and the forward and reverse sequences were assembled using CLUSTALW^83^. The sequences were aligned using MUSCLE v. 3.8^84^. Sequence data are available on Genbank (LC316340–LC316346, LC671974–LC672025).

Two genetic diversity indices (i.e., haplotype diversity and nucleotide diversity) were calculated for each population using Arlequin v. 3.5^85^. The population genetic structures were estimated using analysis of molecular variance (AMOVA)^86^. Pairwise *F*_ST_ between populations was calculated by Arlequin v. 3.5 with 1000 permutations. To visualize the geographical distribution pattern of haplotypes, haplotype networks were reconstructed using a median-joining network^87^ implemented in PopART^88^.

### ddRAD-seq, SNPs detection, and genetic analyses

To prepare a library of double digest restriction site-associated DNA sequencing (ddRAD-seq), 29 genetic samples of *Monodonta* sp. were used (Table 1). RNase was added to the total DNA and it was digested by two restriction enzymes (EcoRI and MspI). P1 and P2 adapters were ligated to DNA fragments according to the ddRAD-seq protocol^89^. The ligated samples were multiplexed and purified with the NucleoSpin gDNA Clean-up kit. Pippin Prep (Sage Science, MA, USA) was used to collect approximately 450 base pair (bp) DNA fragments. The DNA fragments were amplified in eight single PCR reactions, and the products were cleaned using the NucleoSpin gDNA Clean-up kit. The constructed DNA library was sent to Oregon State University’s Center for Genome Research and Biocomputing and sequenced using Illumina HiSeq 4000 single-end sequencing, yielding maximum read lengths of 100 bp. Raw sequence data were deposited in the DDBJ Sequence Read Archive (DRA013313). The raw Illumina sequence reads were demultiplexed and processed with the ipyrad pipeline^90^. If the obtained reads were low in quality or contained adapter sequences, it was trimmed and set to a minimum length of 35 bp using ipyrad’s parameter setting. Reads were clustered at 85% sequence similarity. The minimum number of samples was set to 26/29 (0.896%). Other parameters followed the default setting of the ipyrad pipeline and output datasets for subsequent genetic analysis.

To grasp genetic population structure, we estimated individual genotypes using STRUCTURE v. 2.3.4 according to the ipyrad analysis toolkit (https://ipyrad.readthedocs.io/en/latest/index.html). The number of preassigned genetic clusters (K) was assumed to range from 1 to 10, and seven independent runs were performed for each K value. Each run included 50,000 burn-in iterations and 100,000 iterations. We also performed a principal component analysis (PCA) with GenoDive v. 3.03^91^ and PCAs were plotted by PAST 4.04^92^. Temporal gene flow rates and inbreeding coefficient among four populations were estimated by BA3‐SNPs^93^. The delta values (− m, − a, and − f) selected were 0.3250, 0.5500, and 0.0750, respectively. The number of runs were 30,000,000 MCMC iterations including 6,000,000 burn-ins (20%) and 1000 sampling intervals. Tracer v. 1.6.0 was used to verify the Bayesian convergence and parameter values throughout generations^94^. The AMOVA and pairwise *F*_ST_ between populations were calculated with Arlequin v. 3.5.

The above results of genetic analyses showed that the populations of the northern (Kitanoshima Island and Yomejima Island) and the southern (Chichijima Island and Hahajima Island) islands are genetically differentiated (see “Results” section), and we then estimated its divergence timing by fastsimcoal2 with simplified demographic models (Fig. 5)^95^. To grasp the demographic history of *Monodotna* sp. in the Ogasawara Island, we calculated the two time series: the recovery timing of ancestral population size from the bottleneck that occurred when arriving at the Ogasawara island (t1) and the timing of genetic differentiation (t2) between the northern (Kitanoshima Island and Yomejima Island) and the southern (Chichijima Island and Hahajima Island) islands. The pilot run with a broad range of parameters was conducted and the parameter settings were determined according to the results of pilot run (Table S2). We calculated the site frequency spectrum using easySFS (https://github.com/isaacovercast/easySFS). We carried out 100 independent runs of 100,000 coalescent simulations in each model. Then, we used a simulation with the highest likelihood values for parameter estimation.

## Supplementary Information

Supplementary Information.
