# Supplementary material for: High-throughput SNPs dataset reveal restricted population connectivity of marine gastropod within the narrow distribution range of peripheral oceanic islands
Source: Sci Rep. 2022 Feb 8;12:2119. doi: 10.1038/s41598-022-05026-z (PMC8825847; doi:10.1038/s41598-022-05026-z)
Supplement: Supplementary file 1 — Supplementary Information. [file 41598_2022_5026_MOESM1_ESM.docx]

**Figure S1**. PCA plots for four population of *Monodonta* sp. Colour circles showed each individual. Red indicated population of Kitanoshima Islands (Locality number 1). Green indicated population of Yomejima Island (Locality number 2). Purple indicated population of Chichijima Island (Locality number 3). Yellow indicated population of Hahajima Island (Locality number 4). Colour of convex hulls were corresponded with that of population above mentioned. (a) PC1 and PC2 (b) PC1 and PC3 (c) PC2 and PC3.

**Table S1.** Detailed genetic data of each individuals through the ipyrad pipeline.

| ID | Island | reads raw | reads passed filter | clusters total | read depth | clusters hidepth | hetero est | error est | reads consens | loci in assembly |
| --- | --- | --- | --- | --- | --- | --- | --- | --- | --- | --- |
| t01 | Chichijima Island | 2449182 | 2449172 | 97678 | 25.07393681 | 37056 | 0.013509 | 0.001665 | 34458 | 23688 |
| t02 | Chichijima Island | 1803485 | 1803484 | 85914 | 20.99173592 | 34492 | 0.013949 | 0.001636 | 31627 | 25070 |
| t03 | Chichijima Island | 4683622 | 4683613 | 115270 | 40.63167346 | 46745 | 0.014662 | 0.001617 | 42674 | 31639 |
| t04 | Chichijima Island | 7490907 | 7490893 | 113644 | 65.91542888 | 46451 | 0.016778 | 0.001226 | 42439 | 26579 |
| t05 | Chichijima Island | 10131836 | 10131816 | 138265 | 73.27824106 | 51802 | 0.016831 | 0.00109 | 46884 | 33299 |
| t06 | Chichijima Island | 8939141 | 8939127 | 122525 | 72.957576 | 48672 | 0.016557 | 0.001161 | 44342 | 27771 |
| t07 | Chichijima Island | 1565580 | 1565577 | 76218 | 20.54077777 | 33541 | 0.012912 | 0.001716 | 31260 | 22447 |
| t08 | Chichijima Island | 1946916 | 1946908 | 150538 | 12.93300031 | 41192 | 0.013186 | 0.001994 | 37848 | 29573 |
| t09 | Hahajima Island | 1774856 | 1774848 | 87960 | 20.17789905 | 36585 | 0.014052 | 0.001791 | 33605 | 27178 |
| t10 | Hahajima Island | 6604527 | 6604509 | 118870 | 55.56077227 | 46605 | 0.014747 | 0.001361 | 42906 | 27020 |
| t11 | Hahajima Island | 6382603 | 6382586 | 123567 | 51.65283611 | 46760 | 0.023404 | 0.001434 | 42316 | 26050 |
| t12 | Hahajima Island | 4507735 | 4507714 | 118804 | 37.94244302 | 41900 | 0.022352 | 0.001727 | 38108 | 23515 |
| t13 | Hahajima Island | 2767990 | 2767985 | 96570 | 28.66299058 | 41042 | 0.013023 | 0.001874 | 38044 | 24753 |
| t14 | Hahajima Island | 8792793 | 8792775 | 138779 | 63.35810894 | 49246 | 0.015357 | 0.001112 | 45045 | 27971 |
| t15 | Hahajima Island | 10388536 | 10388506 | 138336 | 75.0961861 | 50354 | 0.016703 | 0.001029 | 45999 | 27452 |
| t16 | Hahajima Island | 10026898 | 10026870 | 147637 | 67.91569864 | 54840 | 0.018715 | 0.001235 | 48966 | 33704 |
| t17 | Yomejima Island | 1983855 | 1983847 | 101297 | 19.58445956 | 39993 | 0.014157 | 0.0021 | 36469 | 27767 |
| t18 | Yomejima Island | 1321098 | 1321095 | 96307 | 13.7175387 | 31212 | 0.015313 | 0.002126 | 28241 | 23036 |
| t19 | Yomejima Island | 1539048 | 1539045 | 90181 | 17.06617802 | 33136 | 0.014457 | 0.001636 | 30367 | 24457 |
| t20 | Yomejima Island | 1050317 | 1050312 | 81203 | 12.93439898 | 28075 | 0.015228 | 0.002073 | 25586 | 20644 |
| t21 | Yomejima Island | 1966691 | 1966686 | 110627 | 17.77763114 | 40618 | 0.014241 | 0.002041 | 36975 | 27907 |
| t22 | Yomejima Island | 6273809 | 6273794 | 125100 | 50.15023181 | 41217 | 0.017095 | 0.000815 | 38438 | 13402 |
| t23 | Yomejima Island | 971540 | 971536 | 66348 | 14.6430337 | 19814 | 0.014298 | 0.001351 | 18329 | 14841 |
| t24 | Yomejima Island | 942184 | 942182 | 66277 | 14.21582148 | 19858 | 0.01343 | 0.001329 | 18402 | 15063 |
| t25 | Kitanoshima Island | 687974 | 687973 | 52390 | 13.13176179 | 17105 | 0.016314 | 0.003154 | 15103 | 12656 |
| t26 | Kitanoshima Island | 6990698 | 6990680 | 117435 | 59.52807936 | 31353 | 0.037711 | 0.001297 | 27107 | 18293 |
| t27 | Kitanoshima Island | 6270862 | 6270843 | 117061 | 53.56901957 | 28028 | 0.022689 | 0.000667 | 25676 | 2086 |
| t28 | Kitanoshima Island | 675370 | 675368 | 60020 | 11.25238254 | 18321 | 0.016136 | 0.003028 | 16460 | 13586 |
| t29 | Kitanoshima Island | 6804620 | 6804604 | 112709 | 60.37320888 | 38321 | 0.023315 | 0.001405 | 33825 | 22837 |

**Table S2.** The parameter settings in fastsimcoal2. Generations were set t2 > t1, population size were set Pop_S > Pop_N, Pop_ANC > Pop_ANC_BOT.

,

| Parameter | Minimum | Maximum |
| --- | --- | --- |
| Pop_N | 1000 | 1.0e5 |
| Pop_S | 1000 | 1.0e5 |
| Pop_ANC | 1000 | 1.0e5 |
| Pop_ANC_BOT | 1000 | 1.0e5 |
| t1 | 100 | 1.0e6 |
| t2 | 100 | 1.0e6 |
| MUTRATE | 1.0e-10 | 1.0e-6 |
| MIG1 | 1.0e-9 | 1.0e-2 |
| MIG2 | 1.0e-9 | 1.0e-2 |
